# Modern Trends in Alternative Proteins and Processing Technologies for Sustainable Food Systems with Antioxidant Implications

**DOI:** 10.3390/antiox15050535

**Published:** 2026-04-24

**Authors:** Young-Hwa Hwang, Abdul Samad, Ayesha Muazzam, AMM Nurul Alam, SoHee Kim, ChanJin Kim, Seon-Tea Joo

**Affiliations:** 1 Institute of Agriculture & Life Science, Gyeongsang National University, Jinju 52828, Republic of Korea; philoria@gnu.ac.kr; 2 Division of Applied Life Science (BK21 Four), Gyeongsang National University, Jinju 52828, Republic of Korea; buzdarabdulsamad@gmail.com (A.S.); ashu2nice@gmail.com (A.M.); alam6059@yahoo.com (A.N.A.); soheelyk@gmail.com (S.K.); ckswls09090@gmail.com (C.K.)

**Keywords:** alternative proteins, meat analogs, novel processing technologies, sustainable food systems, structuring technologies

## Abstract

Alternative proteins and novel processing technologies are crucial to transforming contemporary food systems into ones with lower environmental impact while meeting the rising global demand for protein. Alternative protein sources from plants, microbes, insects, and cultivated cells offer diverse nutritional and techno-functional attributes that can partially or fully replace conventional animal proteins in meat analogs and related products. This review synthesizes the current knowledge on major categories of alternative protein sources, including plant-based ingredients, microbial- and fermentation-derived proteins, insect and other emerging sources, and cultivated (cell-based) meat, with a specific focus on their suitability for structured meat analog applications. Modern structuring and processing technologies are discussed, including the traditional wet and dry extrusion to modern technologies like high-moisture extrusion, high-pressure processing, shear-cell technology, 3D printing, fermentation-based structuring, and enzymatic protein modification. Furthermore, this review critically evaluates product design and quality attributes of meat analogs, including physicochemical properties, sensory performance, nutritional aspects, and safety considerations. This review highlights technological and scale-up challenges, as well as the necessity of multi-criteria optimization in sensory quality, nutrition, sustainability, and affordability, and presents research priorities focused on combining multiple protein sources and advanced processing pathways for next-generation meat analog. This review provides an integrated framework linking protein sources, processing technologies, antioxidant functionality, and sustainability considerations to support the development of next-generation meat analogs. In addition, this review highlights the intrinsic antioxidant potential of alternative proteins, emphasizing the role of bioactive peptides, polyphenols, and structure–function relationships in enhancing oxidative stability and product quality.

## 1. Introduction

Alternative proteins and advanced food processing technologies have emerged as critical strategies for addressing the intertwined challenges of increasing global protein demand, climate change, and finite natural resources. These challenges exist within modern food systems [1]. Protein intake rises with urbanization and incomes, mainly proteins from animal sources [2]. This trend has intensified environmental pressures; conventional livestock production, particularly cattle farming, is associated with substantial greenhouse gas emissions, extensive land and water use, biodiversity loss, and high energy consumption. Serious concerns regarding the long-term resilience and sustainability of current protein supply chains are raised by these impacts. Since a full reassessment of protein sourcing, processing pathways, and distribution systems is necessary to align food production with climate mitigation goals, resource efficiency, and nutritional security, consumer acceptability must also be maintained.

Protein-rich foods or ingredients that can be partially or fully substituted for conventional animal proteins in human diets, while delivering comparable sensory quality, nutritional value, and functional performance, are broadly defined as alternative proteins [3]. This review includes plant proteins (e.g., soy, pea, wheat gluten, pulses, and oilseeds) [4,5], microbial- and fermentation-derived proteins (such as mycoprotein, microalgae, and single-cell proteins) [6], insect-derived proteins [7], cultivated (cell-based) meat [8], and hybrid systems [9,10] that combine multiple protein sources. In addition to the biological origin, alternative proteins can be characterized by their techno-functional characteristics, including solubility, emulsifying capacity, gelling behavior, and water or fat binding, which are crucial to determine their applicability in structured food ap-plications, especially meat analogs [11]. Regarding a systems perspective, alternative proteins should consequently be thought of not as new ingredients, but as empowering components of more resource-efficient, flexible, and resilient food systems.

Simultaneously, new processing technologies can be defined as new or improved unit operations, and structuring methods that go beyond the traditional low-moisture extrusion, mixing, and thermal treatment methods used in plant-based foods [12]. These include high-moisture extrusion [13], shear-cell processing [14], high-pressure processing [15], ohmic and microwave heating [16], pulsed electric field technology [17], three-dimensional (3D) food printing [18], precision fermentation, and enzymatic modification [19]. These technologies are specifically tailored to adjust protein conformation, aggregation behavior, and microstructural organization and thus allow the production of fibrous, anisotropic structures with enhanced juiciness and a meat-like bite, which are key attributes to consumer acceptance of meat analog products. Notably, the pro-processing approaches also have the potential to minimize energy inputs, increase processing yields, agri-food by-products, and circulate bioeconomy ideas in ingredient and product design.

A key issue that affects the quality, shelf life, and nutritional integrity of the food systems based on proteins is oxidative stability. Lipid and protein oxidation can cause the development of off-flavors, discoloration, and the breakdown of vital nutrients, decreasing consumer acceptability [20]. Other protein-based products, especially those high in unsaturated lipids and those that are highly processed are especially susceptible to oxidative degradation. More reactive groups could be further revealed with the help of advanced processing technologies altering protein conformation and microstructure to accelerate oxidative reactions under specific conditions [21]. Thus, the addition of antioxidants, in particular, natural antioxidants like polyphenols, tocopherols, and bioactive peptides, has become a viable option to reduce oxidative stress, improve product stability, and facilitate clean-label formulation strategies. Thus, to develop the next-generation alternative protein products successfully, it is necessary to comprehend and manage oxidative mechanisms.

Against this background, the current analysis seeks to bring together the recent developments in alternative protein sources with modern technologies in structuring and processing along with a specific focus on meat analogs. Meat analogs or alternative meat products are developed to replicate the sensorial, nutritional, and culinary properties of conventional meat, and provide lower environmental footprint and, in certain instances, other health related advantages. Although there has been swift commercial expansion and significant technological advancements, a lot of existing meat analog products still have challenges regarding textural complexity, flavor authenticity, nutritional completeness, clean-label formulation, and cost-effective production on a large scale. In this regard, this review aims to summarize recent advances in key alternative protein sources utilized in the formulation of meat analogs with critically assess on processing and structuring technologies that allow the formation of meat-like structure. Moreover, this review also discusses the key sustainability, regulatory, and consumer-driven issues that are likely to shape the next generation of alternative protein. Despite many studies indicating improvements in alternative proteins and meat analog technology, the results are not always similar across the protein sources, processing, and formulation strategies. The differences in the functionality of raw materials, processing conditions and product design tend to produce contradictory findings with respect to the texture development, nutritional quality, and sensory acceptance. Furthermore, most of the literature on sustainability points out the advantages, but some reports indicate restrictions of processing intensity, refinement of ingredients, and scalability [22]. Thus, the review is characterized by summarizing the recent developments and offering a comparative and critical review of the literature to date, while identifying analytic key limitations, gaps in knowledge, and practical considerations that will be helpful in the development of meat analogs in the future. Unlike previous reviews that primarily summarize alternative protein sources or processing technologies independently, this review integrates protein sources, techno-functional behavior, structuring technologies, and sustainability considerations within a structure–function framework. In addition, comparative interpretation of literature findings is provided, highlighting conflicting results, processing-induced oxidative challenges, and formulation limitations. This approach identifies key scientific gaps and provides a more concrete roadmap for the development of next-generation meat analogs.

## 2. Alternative Protein Sources for Sustainable Food Systems

**Figure 1:**
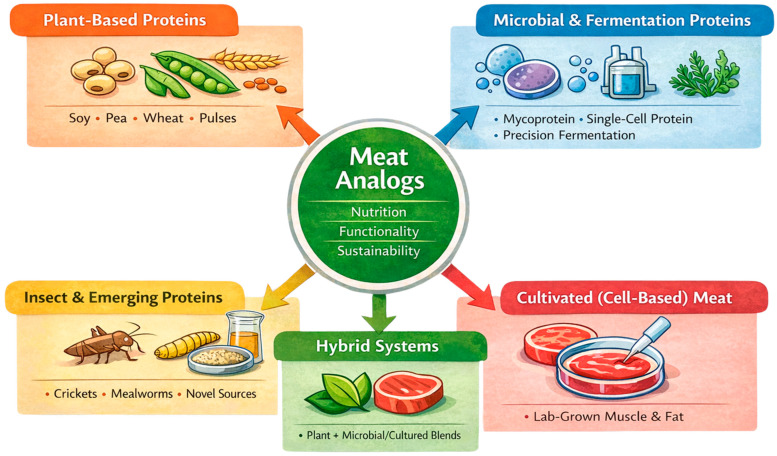
Major types of protein being used as meat analogs.

The major categories of alternative protein sources, along with their nutritional, techno-functional, and sustainability characteristics relevant to meat analog applications, are summarized below in Table 1 and Figure 1. To provide a critical comparison beyond descriptive summaries, different alternative protein sources are evaluated and shown in Table 2 in terms of their textural potential, flavor contribution, scalability, and practical limitations for meat analog applications.

Although various alternative protein sources have been explored for meat analog applications, their practical applicability differs considerably. Plant proteins, particularly soy and pea, remain the most widely used due to their availability, cost-effectiveness, and established functionality in extrusion-based structuring [29]. However, they often require flavor masking and texture optimization. Microbial proteins such as mycoprotein and single-cell proteins offer superior fibrous texture and balanced amino acid profiles, but scalability, regulatory approval, and production cost remain challenges [6]. Insect proteins provide high protein content and functional properties, yet their application in meat analogs is limited by consumer acceptance and labeling concerns [30]. Cultivated proteins represent a promising future approach with potential for authentic meat-like characteristics; however, high production costs, technological complexity, and regulatory hurdles currently restrict their commercial use [8]. Therefore, plant proteins remain the most practically applicable in current meat analog systems, while microbial and cultivated proteins show strong future potential. Moreover, the comparative evaluation of alternative protein sources for meat analogs applications is briefly explained in Table 2.

**Table 2:** Comparative evaluation of alternative protein sources for meat analog applications.

Protein Source	Texture Potential	Flavor Contribution	Industrial Scalability	Key Limitation	Best Application	References
Plant proteins	Moderate to high	Neutral/beany	Very high	Allergenicity	Base matrix	[31]
Microbial proteins	High (fibrous)	Umami	Medium	Cost	Hybrid systems	[32]
Fermentation proteins	Low	Strong flavor	Medium	Regulatory	Flavor enhancers	[33]
Insect proteins	Moderate	Nutty	Low	Acceptance	Protein enrichment	[34]
Cultivated meat	Very high	Authentic	Low	Cost/scale	Hybrid premium	[35]
Hybrid proteins	High	Balanced	High	Formulation complexity	Commercial products	[28]

### 2.1. Plant-Based Protein Ingredients

Plant-based proteins are the primary foundation of meat analogs because of their availability, cost-effectiveness, and versatile techno-functional behavior in complex food matrices [36]. Soy, pea, and wheat gluten, as well as pulses such as lentils, chickpeas, faba beans, and oilseeds—including rapeseed and sunflower—are considered the primary components of plant-based meat analogs [37]. These raw materials are frequently processed into flour, concentrates, and isolates with varying protein contents, particle sizes, and other non-protein components. These characteristics strongly influence their performance in meat analog formulations.

Nutritionally, the differences among plant proteins found in amino acid profile, digestibility and an anti-nutritional factors [38]. Soy and pea proteins are often used as main ingredients in plant based meat analogs because of their protein concentration and balanced amino acid profile [39]. Processing techniques such as soaking, thermal treatment, fermentation and germination are helpful to overcome anti-nutritional compounds and enhance bio-accessibility to enhance the nutritional quality of the final products [40].

From the techno-functional perspective, plant proteins are interesting for their solubility, emulsifying capacity, foaming, gelling, water-holding, and oil-binding properties [41]. These properties collectively define the texture, juiciness, and fat distribution of the meat analogs [42]. Soy and pea proteins have the ability to form heat-induced gels and viscoelastic networks that, under shear in processes such as high moisture extrusion, build anisotropic fibrous structures, which are similar to muscle tissue [43]. Wheat gluten provides excellent elasticity and cohesiveness properties and helps to stabilize the protein matrix to give better bite and likability [44]. Recent comparative studies across diverse botanical sources have shown that the functional properties of plant proteins are highly ingredient-specific and depend on composition, processing history, and environmental conditions, such as pH and ionic strength; thus, careful selection and tailoring of plant proteins for meat analog applications is necessary [45].

### 2.2. Microbial and Fermentation-Derived Proteins

Microbial and fermentation-derived proteins are emerging as key components of sustainable protein portfolios because they offer high productivity, limited land requirements, and the possibility to utilize low-value substrates [6]. Fungal mycoprotein, produced by filamentous fungi in submerged or air-lift fermenters, provides a naturally fibrous biomass with high protein content, dietary fiber, and favorable amino acid composition, and has been commercialized as a meat-like ingredient for several decades [46]. Yeasts and bacteria can be cultivated similarly to produce single-cell protein, often on side streams from agriculture or industry, generating protein-rich biomass suitable for incorporation into food or feed applications [47].

Biomass fermentation refers to processes where the microbial cells themselves constitute the primary product, which can be minimally processed, texturized, or fractionated into protein-rich ingredients for use in meat analogs and other foods [48]. In contrast, precision fermentation utilizes genetically engineered microorganisms to produce specific target molecules, such as heme proteins, dairy proteins, enzymes, or flavor-active compounds, at high titers [49]. These ingredients can be added at relatively low levels to plant-based matrices to improve color development, flavor authenticity, or functionality without dominating texture [50].

In meat analog development, microbial proteins can serve either as standalone bases or as co-ingredients with plant proteins to enhance nutritional quality and sensory complexity [6]. Yeast and bacterial proteins can contribute umami notes and micronutrients [51]. Fermentation applied directly to plant protein substrates can additionally reduce beany off-flavors (beany off-flavors refers to grassy and legume-like undesirable notes commonly associated with plant proteins, particularly soy and pea), modify texture via endogenous or microbial enzymes, and enhance digestibility [52]. Key challenges for wider adoption include production costs, regulatory approvals for novel strains and recombinant products, allergenicity considerations, and consumer perceptions of fermentation-derived foods, but ongoing advances in strain engineering, bioprocess optimization, and downstream processing are rapidly improving feasibility [53].

### 2.3. Insect and Other Emerging Protein Sources

Insect proteins have received significant attention as alternative protein sources because of their high feed conversion efficiency, relatively low greenhouse emissions, and potential for value addition of organic by-products [54]. Commonly farmed species like mealworms, crickets, and black soldier fly larvae contain a high content of proteins, lipids, minerals, and vitamins, and their biomass can be milled to flour, protein concentrates, or isolates, which can be added to different food products [55]. Beyond insects, there are other novel sources, including aquatic plants (e.g., duckweed), microalgae, novel single-cell proteins, and protein-rich fractions recovered from agri-food by-products (e.g., oilseed cakes, brewers’ spent grain, and fish-processing residues) [56]. Valorization of such by-products through physical, enzymatic, or membrane-based fractionation can yield protein concentrates and hydrolysates with enhanced solubility, emulsifying and foaming properties, contributing towards the concept of both sustainability and circular bio-economy [57]. These ingredients can be used to partially replace traditional animal proteins or to complement plant protein matrices in meat analog formulations, thereby enhancing the nutritional density and techno-functional performance of the formulations [58].

However, consumer perception and regulatory issues on food safety is the crucial consideration for emerging sources of protein [59]. Safety assessments of edible insects are mainly concerned with microbiological hazard, chemical contaminants (e.g., heavy metals, pesticides) and allergenic potential [60]. Several species are now approved as novel food in some regions after an intensive risk evaluation [61]. Consumer acceptance studies in Western societies have shown considerable neophobic behavior of consumers, unfamiliarity, and concerns for food safety being significant barriers [62]. Regulatory frameworks for insects, algae, and other novel proteins vary across jurisdictions and often require de-tailed characterization, toxicological data, and transparent labeling, which can delay market entry but also help build consumer trust [63].

### 2.4. Cultivated (Cell-Based) Meat and Hybrid Products

Cultured meat, or cell-based meat, is produced by cultivating animal stem cells in controlled bioreactor conditions [8]. The primary reasons for cultured meat production are to minimize environmental impact by reducing methane emissions, to minimize meat costs so it is economical for consumers, and to provide antibiotic-free meat [64]. The process is usually initiated by isolation of the stem or progenitor cells from an animal, and expansion of these cells in a nutrient-rich culture medium that contains amino acids, sugars, lipids, vitamins, minerals, and growth factors [65]. To produce structured tissue, cells need to be attached to scaffolds or microcarriers that offer mechanical support, porosity, and biochemical cues to enable the formation of muscle fibers, fat depots, and connective tissue in 3D architectures [66].

Scaffolds for cultured meat can be made from consumable biopolymers, such as plant-based proteins, polysaccharides, or decellularized extracellular matrix [67]. Scaffolds can be treated through processing techniques, such as extrusion, freeze structuring, or even 3D printing in order to strengthen anisotropic alignment and texture [68]. Hybrid products blend cultured cells with plant- or microbial-based matrices, with the plant or fungal component providing bulk structure and texture, and the muscle or fat cells providing flavor and aroma to the product [3]. This hybridization approach can reduce the amount of cultivated biomass required and thus, the cost, and overcome the difficulty with scale-up while leveraging existing meat analog processing technology [10].

Despite rapid progress in the cultured meat research still this industry is facing significant hurdles, such as high costs of production, development of media free of animal components, scalability of bioreactors regulatory approval and consumer acceptance [8]. Life cycle assessments (LCA) show that the environmental benefits are greatly affected by the energy sources and the process efficiencies, and thus the need for optimization at the systemic level [69]. Hybrid products with cultured cells, plant proteins, and other alternative protein sources have been proposed as a feasible first step to allow for the progressive introduction to the market and the increment of sustainability [10]. On the other hand, the underlying cell culture and bio-process technologies are progressing apace.

## 3. Modern Structuring and Processing Technologies

Conventional processing of meat analogs has traditionally involved techniques like low-moisture processing (extrusion), texturization, spinning, kneading, and mixing to turn plant proteins into texturized vegetable proteins (TVPs) [70]. TVPs can be hydrated and can be further formulated into burgers, nuggets, and sausages [71]. Low-moisture extrusion (normally <30% moisture) uses single- or twin-screw extruders to impart thermomechanical energy, enabling proteins to undergo denaturation, cross-linking, and expansion to form porous, sponge-like structures [72]. This sponge-like texture has the ability to absorb water and fat during rehydration. These TVPs are being extensively used as this technology is scalable and compatible with soy, wheat, and pea proteins which blends them with starches and fibers.

### 3.1. Spinning and Fiber Forming Methods

Spinning and fiber-forming methods (e.g., spinning protein solutions) have also been studied to obtain aligned protein filaments [73]. Moreover, kneading and mixing are also essential in terms of the distribution of lipids, binders, and seasonings, as well as the formation of the initial dough structure for the production of hybrid meat analogs [10]. However, conventional processes have a number of limitations in terms of development and mimicking the whole-muscle products. Low-moisture extrusion generally produces isotropic or puffed structures with short fibers, resulting in a crumbly or spongy mouthfeel that contrasts with the aligned fibrous structure of muscle tissue [74]. In addition, process conditions can be harsh, leading to excessive protein accumulation, reduced solubility, and off-flavor formation. While there are some methods, such as high-moisture extrusion and alternative structuring technologies, that are more reliable for developing the fibrous architecture of meat and enable the use of a more diverse range of protein ingredients to control heterogeneous structuring at large-scale production.

### 3.2. Innovative Thermal and Non-Thermal Technology

Novel thermal and non-thermal technologies have been presented to overcome the textural and quality limitations of conventional processing to improve the texture for meat analogs. A key development is high-moisture extrusion (HME), which can use the moisture content that is usually greater than 40–50% in conjunction with a cooling die to create dense, fibrous structures more like whole-muscle meat [75]. Under such conditions, proteins are exposed to controlled denaturation, alignment, and phase separation by the shear and temperature gradient [13]. The cooling die suppresses the expansion and fixes an anisotropic microstructure [76]. HME has emerged as one of the most industrially relevant technologies for the production of plant-based steaks, filets, and strips because of its capability of providing layered textures and improved juiciness as compared to low-moisture extrusion [77]. High-moisture extrusion produces anisotropic fibrous structures suitable for whole-cut analogs, whereas low-moisture extrusion is more appropriate for ground meat analogs [78]. However, high-moisture extrusion requires higher energy input and specialized cooling dies, which may increase processing complexity. In contrast, shear-cell technology provides well-aligned fibers with precise structural control but remains largely limited to pilot-scale applications. These differences suggest that each structuring technology presents specific advantages and limitations, and their suitability depends on desired product characteristics, scalability requirements, and processing considerations [79].

Non-thermal or minimally thermal technologies, such as high-pressure processing (HPP) [80], pulsed electric fields (PEFs) [17], ohmic heating [16], and microwave heating [81], are being explored to alter protein structures, enhance safety, and fine-tune texture with less thermal damage. HPP can cause protein gelation and network formation at relatively low temperatures, which can improve firmness and water holding properties in meat analog matrices [82], as well as destroy pathogens [83]. PEFs and ohmic heating allow fast, volumetric input of energy and may affect the conformation and phase behavior of the protein [84], which presents opportunities to restructure, or can even be used as a pre-treatment before the extrusion process. Microwave heating can provide fast and uniform heating in formed products, which affects expansion, porosity, and the final texture [85]. Although many of these non-thermal methods are still at pilot scale for meat analogs but they have potential for reducing energy consumption, product quality improvement and enabling new texture archetypes by extrusion or other thermo-mechanical processes.

### 3.3. Structuring Approaches Based on Shear and Advanced Ones

Shear-based technologies such as high-moisture shear-cell processing have emerged as promising technologies as alternatives or complimentary to extrusion in producing fibrous plant-based meat analogs [14]. In shear-cell technology, a protein rich dough is subjected to a controlled and uniform shear at elevated temperatures, which promotes alignment and aggregation of proteins to anisotropic structures, without the complex flow patterns which occur in extruders [86]. This method is relatively low in throughput but provides the opportunity to precisely control shear rate, temperature, and residence time, often requiring less energy input and simpler equipment compared to extrusion. Studies reveal that shear-cell processing was able to produce well-defined fibrous structures from the systems of soy, pea, and mixed proteins, especially when combined with appropriate lipids and hydrocolloids [86].

### 3.4. 3D Printing

3D food printing (additive manufacturing) has emerged as an advanced structuring technique for plant-based meat analogs [87]. In this method, protein-based inks composed of plant proteins, oils, water, and hydrocolloids are deposited layer-by-layer through a nozzle to form designed structures [88]. The technology allows precise control over the spatial distribution of ingredients, enabling the creation of complex internal architectures, anisotropic textures, and customized shapes. By adjusting printing parameters such as nozzle diameter, extrusion rate, and printing speed, the microstructure and mechanical properties of the printed product can be tailored. Studies have shown that plant protein formulations based on soy, pea, or mixed proteins can be successfully used as printable inks to produce meat-like textures and soft fibrous structures [89]. Furthermore, 3D printing enables the fabrication of structured products with controlled composition and texture variations within a single product, making it a promising approach for next-generation meat analogs [90].

### 3.5. Fermentation and Enzymatic Modification as Structuring Means

Fermentation and enzymatic modification is powerful biological method for the customization of the structure and activity of proteins for meat analogs [91]. Fermentation with the aid of specific microorganisms (e.g., lactic acid bacteria, yeasts, filamentous fungi) can change the structure of plant protein matrices [92]. Fermentation in meat analogs can improve water retention, emulsification, and gelation, thus improving texture and juiciness, and at the same time reduce undesirable beany off-flavors and produce appealing meaty tastes due to the modification of amino acids and lipids [91]. The fermentation of extruded protein products or textured vegetable proteins has been shown to enhance the structural integrity, chewiness, and the overall sensory acceptance of plant-based meat prototypes [87].

Overall, each structuring technology presents distinct advantages and limitations. High-moisture extrusion is currently the most industrially scalable approach for producing fibrous meat analogs, whereas shear-cell technology offers precise structural control but remains limited to pilot-scale production [93]. 3D printing enables customization and complex structures but suffers from low throughput and high production cost [94]. Non-thermal technologies such as high-pressure processing and pulsed electric fields have potential for texture modification; however, their industrial application in meat analogs remains limited [90]. Therefore, the selection of structuring technology depends on balancing product quality, scalability, and economic feasibility.

## 4. Product Design and Quality of Meat Analogs

Product design for meat analogs combines the choice of the ingredients, structural technology, and formulation methods to develop a product that imitates the conventional meat product in terms of its appearance, texture, flavor, nutrition, and handling, ensuring that the sustainability and regulatory requirements are met [10]. Key quality factors in plant-based meat analogs encompass physicochemical and structural attributes, sensory performance and consumer perception, nutritional and health aspects, as well as safety, shelf life, and packaging. Particular attention is given to the interactions among protein sources, lipids, binders, and hydrocolloids, and to the compromises involved in simultaneously achieving meat-like texture, clean labels (clean label refers to formulations with simple, recognizable ingredients, minimal use of artificial additives, and improved nutritional profiles).

### 4.1. Physicochemical and Structural Characteristics

The physicochemical and structural properties of meat analogs influence their appearance, texture, cooking properties as well as their overall consumer experience [10]. The microstructural arrangement and orientation of protein fibers, pores, and fat inclusions determine the anisotropy, juiciness reservoirs, and the fracture behavior during mastication. The water- and fat-binding properties of the protein matrix influence yield, cooking loss, and perceived juiciness, while higher water-holding capacity is generally associated with improved tenderness and flavor. Texture profile factors, such as hardness, cohesiveness, chewiness, and springiness, are manipulated by various means, including protein source, process variables (high-moisture extrusion, shear cell, etc.) and post-processing techniques (chilling, storage, etc.) [95]. Color in plant based meat analogs is generally produced utilizing natural colorants (e.g., beetroot, carotenoids, legume derived heme analogs) [96] and regulated Maillard processes to replicate the appearances of raw and cooked meat [97]. Lipids, binders, and hydrocolloids fulfilled essential functions. Plant oils and structured fats have an influence on lubricity and dispersion of taste. Binders such as isolated proteins, starches and fibers have ability to improve the matrix integrity. Hydrocolloids (e.g., methylcellulose, carrageenan) for gelation, water retention and thermal stabilization [58]. The careful utilization of these technologies, along with accurate ingredients, have ability to produce products to resemble fresh meat in texture and appearance, as well as process ability and stability.

Texture development in meat analogs is strongly influenced by protein type and processing conditions. For example, high-moisture extrusion of soy protein isolate has been shown to produce aligned fibrous structures with increased hardness and chewiness compared with pea protein-based formulations, which often result in softer textures due to lower gel strength [98]. Similarly, the incorporation of wheat gluten improves elasticity and fibrousness because of its viscoelastic network-forming ability [43]. Water-holding capacity is also affected by protein functionality and fiber addition; Bakhsh et al. [99] reported that the inclusion of methylcellulose or dietary fibers enhances water retention and reduces cooking loss in plant-based meat analogs. In addition, structured fat systems such as emulsified vegetable oils or oleogels have been shown to improve fat distribution and mouthfeel, contributing to juiciness and overall sensory acceptance [100].

**Figure 2:**
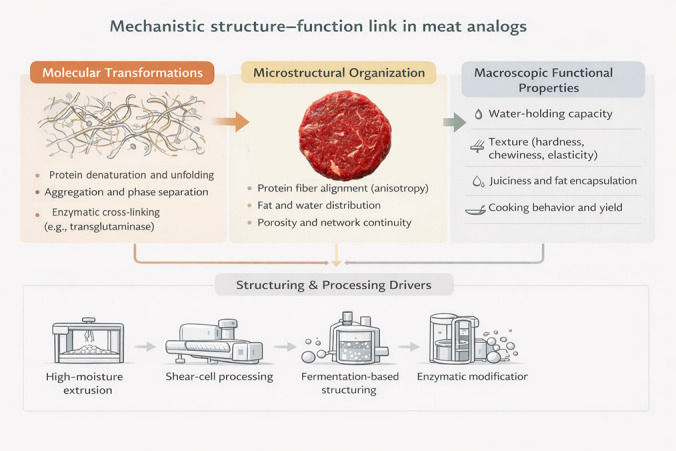
The relationship between protein structuring, microstructural organization, and macro-scopic quality attributes in meat analogs. This figure illustrates the hierarchical structure–function relationship in meat ana-logs, linking molecular transformations to functional properties. At the molecular level (left), protein denaturation, aggregation, and enzymatic cross-linking drive structural modifications. These changes lead to the formation of microstructural features (center), including protein fiber alignment, phase distribution of fat and water, and network continuity. In turn, these structural attributes determine macroscopic functional properties (right), such as water-holding capacity, texture (hardness, chewiness, and elasticity), juiciness, and cooking behavior. Arrows indicate the directional progression across hierarchical levels. The lower panel highlights key structuring and processing drivers—such as high-moisture extrusion, shear-cell processing, fermentation-based structuring, and en-zymatic modification—that influence transformations at all levels. Color coding distinguishes each hierarchical stage, while icons represent major processing technologies.

Key physicochemical and structural quality characteristics along with formulation and processing strategies of meat analogs are further summarized in Table 3. The relationship between protein structuring, microstructural organization, and macroscopic quality attributes in meat analogs is schematically illustrated in Figure 2.

### 4.2. Sensory Properties and Consumer Perception

Taste and aroma are the decisive factors in consumer acceptance of meat analogs [106]. Flavor generation is reliant on the precursor of amino acids, reducing sugars, and lipids, for Maillard and lipid oxidation reactions during cooking which is further supported by yeast extract [107]. Fermented ingredients and natural flavors provide umami flavor to meat analog [108]. Different strategies, e.g., selection of raw materials, solvent or steam stripping, fermentation, and the use of flavor modulators, are needed to overcome beany, grassy, or bitter off-notes usually linked to some plant proteins [109]. Juiciness depends on the inclusion of water and fat-binding agents, emulsified or encapsulated fats, and thermal gelling systems for moisturizing and releasing oil within the product [110]. Consumer perception of alternative proteins and meat analogs is not only affected by sensory performance, but also by beliefs, labeling and attitude towards processing [36]. Drivers of acceptance include taste resemblance with meat, health, and environmental benefits. While the barriers include unfamiliar ingredients, concerns about ultra-processed foods and high prices [111]. For consumer acceptance, effective product design in terms of optimized sensory attributes, along with awareness among consumers and clean labeling strategies, is highly important. Moreover, consumer acceptance of meat analogs is not uniform across populations. Younger consumers and flexitarians often emphasize sustainability and health benefits, while older groups prioritize taste resemblance and texture familiarity [112]. Regional cuisines also shape expectations, with stronger umami favored in Asian markets and smoky or grilled notes preferred in Western contexts. Moreover, cultural attitudes toward processing, labeling transparency, and price sensitivity significantly influence acceptance [113]. Considering these demographic and regional differences is essential for tailoring product design and marketing strategies

### 4.3. Nutritional Quality and Health

The nutritional quality of meat analogs depends on the amount and quality of protein, amino acid composition, digestibility and overall nutrient composition, which includes fats, carbs, fiber, vitamins, and minerals [114]. Combining complementary plant proteins and microbial or other alternative proteins can enhance necessary amino acid profiles as well as strengthen adequate protein quality metrics [115]. Processing techniques such as heat treatment, fermentation and enzymatic modification can reduce anti-nutritional factors and improve the digestibility of foods; however, too much thermal or oxidative damage can reduce the functionality of certain amino acids [116]. Decisions about the addition of different types and amount of fat in meat analogs is fundamental, as there is an opportunity to replace animal saturated fats with structured plant unsaturated oils which improves the lipid profile of the final product [117]. Meanwhile, total fat and energy density must also be considered when producing meat analogs. The use of salt and additives is essential for the effectiveness of technologies and health benefits for the consumers. Meat analogs often rely on the use of sodium for their flavor and function, and this creates a clash with public health recommendations and texturizing [87]. The preserving chemicals in meat analogs can raise fears about clean labeling [118]. Fortification that includes iron, vitamin B12, zinc and other micro nutrients can overcome deficiencies in consumer which makes meat analogs good for health [119]. Overall, nutrition design should be done in an approach of multi-criteria optimization by balancing protein quality, lipid composition, sodium level, calorie density, and fortification within the regulatory and sensory limitations. Furthermore, Table 4 shows a comparative analysis of the nutritional characteristics and health implications of meat analogs in comparison to conventional meat. Beyond macronutrient composition, the bioavailability of fortified micronutrients such as iron, zinc, and vitamin B12 remains a challenge, as plant matrices often contain phytates and other compounds that inhibit absorption [120]. Comparative studies confirm that, despite higher iron content in some plant-based burgers, absorption is reduced relative to beef [121]. Moreover, despite their nutritional advantages, many meat analogs are classified as ultra-processed foods, raising concerns about long-term health implications and consumer trust [122]. Balancing nutritional quality with clean labeling, reduced sodium, and improved micronutrient bioavailability is therefore essential for the sustainable development of meat analogs.

### 4.4. Safety, Shelf Life, and Packaging Issues

The safety and shelf life of meat analogs are determined by microbiological stability, chemical stability, and packaging efficacy [127]. The microbiological safety is dependent on the quality of raw materials, hygienic processing and the use of suitable thermal or hurdle treatments [128]. Although plant-based matrices are free of animal-associated diseases, nonetheless, they can enable the growth of rotting things and general foodborne pathogens if not properly managed [129]. Allergenicity is an important issue, as there are many sources of proteins (e.g., soya, wheat, pea, nuts) identified as potential allergens, and thus they need to be explicitly labeled and carefully formulated for susceptible people [130]. Anti-nutritional factors and potential process pollutants, such as acrylamide and advanced glycation end-products, need to be monitored, especially in cases of severe heat processing. Oxidative stability is a very important issue as unsaturated plant oils and added flavors are prone to lipid oxidation which can cause off-flavors, color changes, and unfortunate nutritional degradation [131]. These effects can be alleviated with the use of antioxidants (natural extracts and tocopherols), oxygen-barrier packaging, and changed atmospheres. Packaging techniques are focused more and more on combining high barrier performance with recyclability or biodegradability, as well as taking into account cold chain or ambient stable products, depending on the formulation [132]. The effective methods of safety, shelf life, and packaging requires the integration of the selection of ingredients, process parameters, and storage conditions in order to ensure that meat analogs remain safe, high quality, and sensory acceptable throughout their designated shelf life.

**Figure 3:**
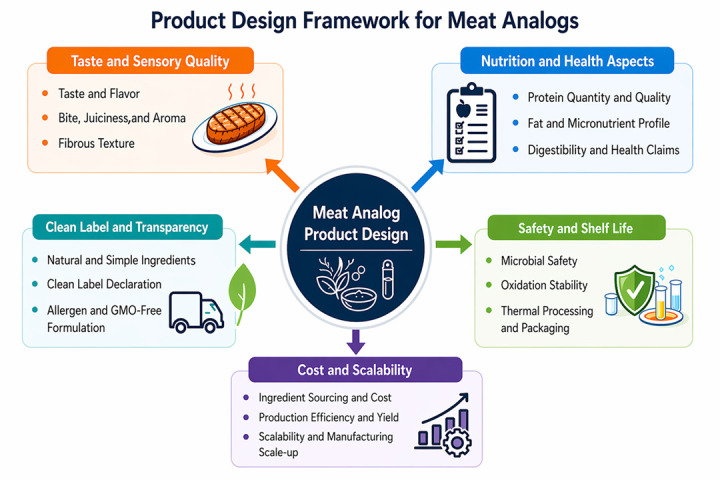
Comprehensive product design framework for meat analogs illustrating key considerations including sensory quality; nutritional value; safety and shelf life; clean-label attributes; and cost and scalability.

Beyond microbiological and oxidative stability, plant-based meat analogs face risks of pathogen contamination and spoilage driven by diverse microbial communities [133]. Effective control requires strict hygienic processing, hurdle technologies, and rapid microbial testing. Shelf life can be extended through modified atmosphere packaging, vacuum sealing, and active packaging with oxygen scavengers or antimicrobial films [132]. Chemical safety concerns such as acrylamide, AGEs, and PAHs formed during high-temperature processing must be monitored and minimized. Emerging packaging innovations, including biodegradable films, nanocomposites, and smart indicators, not only enhance safety and shelf life but also align with sustainability and consumer trust requirements. A comprehensive product design framework, which takes into account the sensory, nutritional, safety and technological considerations for meat analogs, is shown in Figure 3.

### 4.5. Lipid Oxidation and Antioxidant Strategies in Meat Analogs

Lipid oxidation is one of the most critical quality parameters affecting plant-based meat analogs (PBMAs). Unlike traditional meat, plant-based products contain high levels of polyunsaturated fatty acids that are particularly susceptible to auto-oxidation. This process leads to the generation of reactive oxygen species, biogenic amines, malonaldehyde (MDA), and other oxidation products that are detrimental to human health [134]. Different plant-based meat alternatives show varying susceptibility to lipid oxidation. Research comparing seitan, tempeh, and tofu found that tofu exhibited significantly higher lipid oxidation levels, as well as a dramatic 92.2% depletion of naturally occurring antioxidant tocopherol following processing [135]. In contrast, processing practices used in tempeh and seitan manufacturing are more promising strategies for limiting oxidation and Maillard reactions in food.

Plant-based antioxidants include polyphenols, flavonoids, tannins, terpenes, alkaloids, saponins, and coumarins—all possessing significant antioxidant and antimicrobial properties [134]. These bioactive compounds can reduce the rate of auto-oxidation and microbial growth, thereby extending the shelf life of meat products when added under appropriate conditions and concentrations (0.025 to 2.5% *w*/*w*). Green tea extracts demonstrate remarkable effectiveness in reducing lipid oxidation in plant-based patties throughout 28-day storage periods [136]. The extracts’ L-theanine and tannin contents influence both taste and physicochemical properties, with extracts containing higher tannin levels showing enhanced emulsion stability and texture improvements through non-covalent interactions between tannin and pea protein.

Phenolic extracts from olive mill by-products, rich in oleacein, verbascoside, and hydroxytyrosol, maintain stable phenolic concentrations throughout shelf life and significantly enhance antioxidant activities in plant-based meat analogs composed of lentils and mushrooms [137]. Similarly, both carrot and tomato powders increase total phenolic content and antioxidant stability, with tomato powder demonstrating superior efficacy in inhibiting lipid oxidation in soy protein-based meat patties [138]. Nanoemulsions of *Litsea cubeba* and cinnamon essential oils, when emulsified with chitosan and Tween 80, exhibit remarkable stability with droplet sizes of approximately 5 nm and zeta potential of 95.13 ± 2.67 mV [139]. When applied to PBMAs stored at 4 °C, these nanoemulsions significantly reduced thiobarbituric acid reactive substances (TBARS), inhibited pathogenic bacteria growth, and preserved sensory quality.

Rosemary, oregano, thyme, and clove essential oils exhibit strong antimicrobial and antioxidant properties, inhibit lipid oxidation, and prolong the shelf life of meat under refrigerated conditions [140]. These bioactives in plants combine to act via several different mechanisms: suppression of spoilage and pathogenic bacteria, prevention of oxidative degradation, and preservation of product color and sensory properties. Recent developments utilize nanoemulsions, electrospun nanofibers, biopolymer coatings, and smart packaging to deliver controlled release of these bioactives. Sustained release lipo-some-based dual antioxidant delivery systems have great potential, and lipophilic resveratrol and hydrophilic gallic acid were both successfully encapsulated into liposomes, which inhibited the formation of lipid hydroperoxides and MDA, preserved the color, and reduced the production of oxidation products [141]. The dual delivery approach takes advantage of complementary antioxidant mechanisms

Recent studies show that a combination of various methods of stabilization is better. A synergistic system using glucose oxidase (GO), phytase (PA), and tamarind gum (TG) of high-moisture extruded plant-based meat demonstrated that the GO + TG system most effectively inhibited oxidation of proteins, reduced sulfhydryl loss and maintained protein structures during repeated freeze–thaw cycles [142]. Such a two-network system, in which GO enhances covalent cross-linking and TG provides steric stability, shows strong potential for industrial applications. Addition of seaweed about 40 percent is effective to inhibit the migration of water and minimize the oxidative damages by increasing the total phenolic content and free-radical scavenging activity of plant-based meat products [142]. The polysaccharides of the algae create protective nets around the proteins, enhancing both water-binding capacity and antioxidant defense.

The evaluation of lipid oxidation should be properly conducted with the selection of the methodology according to the type of products. Primary oxidation products (hydroperoxides) can be primarily measured by peroxide value and conjugated diene methods, whereas secondary oxidation products are measured by thiobarbituric acid reactive substances (TBARS) and chromatographic methods [143]. More sophisticated methods, such as calorimetric methods (differentiated scan calorimetry and isothermal calorimetry), provide real-time, quantitative determination of antioxidant activity through direct measurement of heat produced during the oxidation process [144]. Such methods are used to assess oxidative activity and provide insight into antioxidant behavior, including synergistic or pro-oxidant effects at various concentrations, which is vital for optimizing preservation strategies.

Commercially produced plant-based chicken schnitzels exhibit a wide lipid stability in cooking under recommended conditions, and total oxidation (TOTOX) values do not show any significant changes between raw and cooked conditions [145]. Likewise, refrigerated storage at the recommended conditions maintains the lipid stability of plant-based nugget and sausage mimics until their best-before dates [146], but texture and color stability need to be considered. Adzuki bean protein proves to possess natural antioxidant activity (maximum phenolic content up to 222.6 μmol GAE/g) and provides natural red coloring and oxidative stability in a period of one year at 25 °C of storage [147]. This inherent bioactivity will minimize the use of synthetic antioxidants and will place such proteins in the position as clean-label solutions.

Berry pomace is a juice and wine waste product that offers antioxidants and natural colorants, as well as dietary fibers that improve nutritional profiles and shelf stability of plant-based meat analogs [148]. Combinations of several preservation strategies, such as green extraction processes (supercritical CO_2_, ultrasound-assisted techniques), nanotechnology use, and synergistic antioxidant formulations, are at the forefront of lipid oxidation control in the context of plant-based meat analogs. The need to solve challenges of variability in compositional properties of natural extracts, cost of production and regulatory factors will be critical in implementation on a large scale basis [140].

Processing technologies have a potent effect on oxidative processes in plant-based meat analog. The endogenous antioxidant tocopherols can also be degraded by thermal processes like extrusion, alkalining, and high-temperature cooking that stimulates lipid oxidation by enhancing the formation of free radicals, destruction of endogenous antioxidants, and unsaturated lipids exposure [149]. Non-thermal technologies, such as high-pressure processing and pulsed electric fields can mitigate oxidative damage by reducing thermal destabilization, but structural destabilization of protein-lipid matrices by oxidation-prone agents can still impact oxidation susceptibility [150]. To this extent, choice of processing conditions is important in trade-off between texture formation and oxidative stability of plant-based meat analogs.

The stability of color and flavor in plant-based meat analogs is strongly connected with oxidative reactions [151]. The natural red colorants like beetroot extract, anthocyanins, and leghemoglobin and other heme analogs are prone to oxidation during processing and storage that causes discoloration and low consumer acceptance [152]. Lipid oxidation products can accelerate pigment degradation, leading to browning or fading of red color [153]. Incorporation of antioxidants such as phenolic compounds, tocopherols, and plant-derived bioactives improves both oxidative stability and color retention [154]. Thermal processing methods such as extrusion and cooking may further promote pigment oxidation, whereas optimized processing conditions and antioxidant incorporation help maintain desirable meat-like redness and visual quality. Furthermore, role of antioxidants in improving oxidative stability, shelf life, and color retention in meat analogs is discussed in Table 5. Additionally, a wide range of exogenous antioxidant strategies have been developed to control lipid oxidation in meat analog systems, and increasing attention is being given to the intrinsic antioxidant capacity of protein ingredients themselves. In this context, alternative protein sources such as plant, microbial, and insect proteins not only contribute to structural and nutritional properties but also serve as natural sources of bioactive antioxidant compounds. However, variability in natural extract composition, potential sensory impacts, and cost considerations remain key challenges for large-scale industrial application. Moreover, these aspects are comprehensively discussed in the following section.

### 4.6. Antioxidant Potential of Alternative Protein Sources

The new sources of protein are increasingly considered both sustainable and nutritious, and they possess antioxidant properties. These alternative protein sources are associated with bioactive compounds such as polyphenols, carotenoids, vitamins, and bioactive peptides [158].

#### 4.6.1. Plant-Based Proteins as Polyphenol-Rich Antioxidant Sources

Plant protein ingredients, especially those derived from legumes, are associated with natural antioxidants because legumes are rich in polyphenols such as flavonoids and phenolic acids. There is also a big variation in the number of plant species as the total phenolic content (TPC) varies widely. Beans were reported to have up to 425.19 mg GAE/100 g, whereas lentil species reported 69 to 85.89 mg GAE/100 g [159]. The compounds are mainly concentrated in seed coats and are determined by genetic, environmental, and processing factors. Mung beans exhibit flavonoid concentrations of 45.47 mg QE/100 g. Pea protein ingredients derived from biofortification or specific processing strategies may retain or concentrate polyphenols (10.12 ± 0.27 mg GAE/g in processed products), contributing to enhanced antioxidant capacity relative to conventional plant protein ingredients [160]. The processing technologies have a significant impact on the antioxidant profiles, as per similar study done by Amanipour et al. [161] reported that the fermentation of gray pea flour has led to an incredible increase in total phenolic content by 67% and total antioxidant capacity by FRAP and DPPH assays up to 104%. Similarly, fermentation of lentils by *Aspergillus oryzae* and *A. niger* strains in solid states yielded greater antioxidant activity via FRAP and DPPH with increased values of up to 107% and 81%, respectively [162].

Isoflavones, including genistein and daidzein, are especially abundant in soybeans and soy-derived ingredients and are potent antioxidants due to various mechanisms, including free radical scavenging and metal ion chelation, and have been extensively investigated to prevent cardiovascular diseases and inflammation-related pathologies [163]. Lentils are an underestimated source of bioactive compounds, as they are not only rich in phenolic acids and flavonoids but also tocopherols and carotenoids, and a full spectrum of phytochemical activity has been observed to support antioxidant and anti-inflammatory effects due to enzymatic and non-enzymatic antioxidant systems [164]. The consumption of lentils has been linked to lower morbidity rates of various chronic diseases as a result of the synergistic effect of various polyphenolic compounds and their metabolites [165]. Pea (*Pisum sativum*) is rich in polyphenols, including flavonoids and phenolic acids, which contribute to its antioxidant potential, with flavonoid and phenolic acid as the most abundant bioactive compounds that confer antioxidant, anti-inflammatory, and antidiabetic effects, and new technologies in protein enrichment have resulted in pea-based products with significantly higher polyphenol levels approaching those of medicinal plants [166].

#### 4.6.2. Microbial Proteins: Engineered Antioxidant Sources

Some of the algal species are also being used in the formation of meat analogs [167] as algae are a renewable and sustainable source of proteins that have extraordinary antioxidant properties due to their biosynthesis of specialized metabolites, such as carotenoids, glutathione, and phenolic compounds. Microalgae, including *Scenedesmus* spp. and other green algae, synthesize proteins rich in tocopherol and carotenoid precursors; similarly, marine microalgae of the genus *Caulerpa* produce bioactive compounds such as caulerpin and caulerpinic acid, exhibiting comparable intracellular antioxidant properties [168,169]. The content of algal proteins is 80 to 120 mg of GAE equivalent/100 g of total phenolic compounds, with astaxanthin and fucoxanthin being particularly strong antioxidant carotenoids [170], and these carotenoid pigments act via singlet oxygen quenching and lipid peroxidation inhibition to photooxidative stress and free radical-mediated cellular damage [171].

During growth, yeast species, especially *Rhodotorula* and *Phaffia* spp., store large amounts of carotenoids, thus becoming appealing cellular factories to produce bioactive metabolites. The carotenoid-rich (beta-carotene, torulene, torularhodin) yeast proteins are known to contain not only nutritional amino acids but also lipid-soluble antioxidants that partition into cellular membranes and help prevent peroxidative damage [172]. Although they have high antioxidant potential, the high cost of production, regulatory acceptance, and inconsistency in metabolite composition continue to limit the scale of application of microbial proteins.

#### 4.6.3. Insect Proteins: Emerging Sources of Antioxidant Peptides

Edible insects are a new source of protein in meat analog applications owing to their excellent protein quality and bioactive compounds. Traditional species like *Acheta domesticus*, *Tenebrio molitor*, *Locusta migratoria*, and *Bombyx mori* store 48–67% protein (dry weight basis) and have equal mixes of essential amino acids that can be utilized in food creation [173]. Besides protein content, insect-based ingredients are also sources of polyphenols, lipids, and chitin-based products, which are antioxidants and functional. Total phenolic content is reported to range between 150 and 250 mg GAE/100 g of crickets, and 120–220 mg GAE/100 g of mealworm and silkworm proteins [174].

Bioactive peptides with high antioxidant activity are enzymatic hydrolysates of insect proteins, which have a high relevance in enhancing oxidative stability in meat analog systems. Mealworm protein hydrolysates also possess multifunctional bioactivity such as antioxidant potential and enzyme inhibitory effects that can be used to enhance the functionality and nutritional benefits of hybrid or plant-based meat products [175]. Also, insect proteins are the good source of the necessary lipids, especially the polyunsaturated fatty acids, which can enhance the nutritional value but have to be stabilized by antioxidants because they are sensitive to oxidation [176].

Although antioxidant activity of alternative protein sources is frequently described as the total amount of phenolic compounds or radical scavenging activities, their usefulness in meat-analog systems is highly reliant on structure–function relationships and processing interactions. The conformation of the protein, the size of the peptide, the amino acid composition, and the association with other food matrix components are the factors which determine the effectiveness of these antioxidant compounds. As an illustration, peptides with low molecular weight which are the products of protein hydrolysis have enhanced radical scavenging and metal-chelating properties because of the presence of amino acids like histidine, cysteine, and tyrosine. Nevertheless, thermal treatment processes like extrusion have the ability to increase and decrease antioxidant activity with the intensity of processing. Moreover, the synergistic effects of the protein and co-existing substances (e.g., polyphenols, carotenoids, and lipids) can have a substantial effect on oxidative stability in complex systems (e.g., meat analogs). Thus, in addition to compositional analysis, a mechanistic knowledge of the action of these bioactive components in structured food matrices is required to maximize performance of antioxidants and techno-functional characteristics. Despite their promising antioxidant potential, the practical application of alternative protein-derived antioxidants in meat analog systems is influenced by processing stability, interactions with other ingredients, and regulatory and cost constraints. Therefore, translating these functionalities from laboratory findings to industrial-scale applications remains a key challenge.

## 5. Sustainability, Circularity, and Systems Perspective

**Figure 4:**
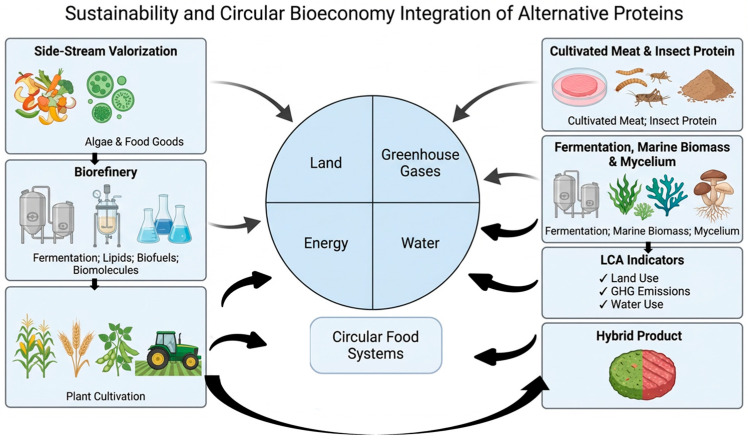
Integration of alternative proteins into circular and regenerative food systems, demonstrating side-stream valorization, biorefinery pathways, and sustainability assessment based on life cycle indicators, such as land use, greenhouse gas emissions, and water use.

Alternative proteins and their processing need to be evaluated in more extensive systems considering the environmental and circularity of resources, as well as socio-economic and ethical aspects. The interactions of meat analogs with other alternative protein systems and environmental resources (climate, land, and water) present significant opportunities for advancing circular and regenerative food systems [177]. Policy, ethics, and governance serve as fundamental pillars in supporting and accelerating the transition toward sustainable food systems. The integration of alternative proteins within circular and regenerative food systems is illustrated in Figure 4, highlighting their roles in side-stream valorization, bio refinery processes, and sustainability assessment through key environmental indicators.

This figure illustrates the integration of alternative proteins within a circular bioeconomy, linking side-stream valorization, biorefinery processing, and alternative protein production to hybrid food development. Sustainability indicators including land, energy, water, and greenhouse gas emissions are evaluated through LCA metrics. The system operates as a closed-loop circular food system to enhance resource efficiency and reduce environmental impact. In this figure, the central circle represents key resource domains (land, water, energy, and greenhouse gases) within circular food systems. Arrows indicate directional flows and interconnections among system components. Color shading distinguishes different system elements and functional categories.

### 5.1. Environmental Impacts and Life Cycle Assessment

Life cycle assessment (LCA) studies consistently show that plant-based meat analogs and other forms of alternative proteins generally have significantly lower greenhouse gas emissions, land use, and water use than conventional beef, pork and poultry, primarily due to lack of enteric methane and lower feed conversion losses [178]. A recent comparative LCA showed that plant-based meats had, on average, 91% lower environmental impacts than beef, 88% less than pork, and 71% less than chicken across multiple environmental categories, including climate, land, and water indicators [178]. Similar studies in individual burger patties have reported that global warming potential and water consumption can be reduced by 65% and 45%, respectively, when a plant-based burger replaces a beef burger under UK conditions [179]. While LCAs consistently show that plant-based meat analogs reduce greenhouse gas emissions and land use compared to beef, results vary depending on methodological choices. Differences in system boundaries [179], allocation methods for co-products [180], and energy inputs during extrusion and refrigeration can significantly influence outcomes. For example, the inclusion of cooking energy or reliance on fossil-based electricity can offset some environmental gains. Harmonized methodologies and transparent reporting are therefore essential. Beyond environmental indicators, integration of meat analogs into circular food systems requires valorization of side streams and biorefinery approaches [181], while socio-economic and ethical dimensions, including affordability and cultural fit, must also be considered [122]. Policy and governance frameworks will play a central role in ensuring that alternative proteins contribute to regenerative and sustainable food systems. Moreover, meat analogs in comparison with conventional meat products for some important environmental indicators are summarized in Table 6.

However, environmental performance is sensitive to factors such as the sourcing of crops, balancing nutrition, processing intensity of ingredients and packaging along with distribution systems. LCAs of meat substitutes suggest that more energy is needed for highly processed products [178]. Still, the environmental effects are often less than that of traditional meat per unit of protein or serving. Although many studies report reduced greenhouse gas emissions for plant-based meat analogs, results vary depending on system boundaries, ingredient sourcing, and processing intensity. Therefore, sustainability benefits should be interpreted cautiously, particularly for highly processed formulations.

### 5.2. Circular and Regenerative Food System Integration

Alternative proteins have a role to play in building circular and regenerative food systems through the valorization of agricultural and food processing by-products as inputs for protein and ingredient production [185]. By-products, e.g., oilseed cakes, cereal bran, brewers’ spent grain, and crop residues, can be fractionated into protein concentrates, hydrolysate, and fermentable sugars, which are used as substrate for plant-based ingredients, fermentation-derived proteins, or cultivated meat media [186]. Side streams identified significant potential for the use of these residue for protein concentrates, protein hydrolysates, and lignocellulosic sugars through the reduction in waste, diversification of revenue streams for farmers, and reduction in net environmental burdens [187].

The integration of alternative protein production with biorefineries enables the sequential utilization of biomass, where high-value fractions such as proteins and nutraceuticals are first extracted, followed by the use of remaining components for energy or material applications. This integration can result in a reduction in greenhouse gas emissions and an enhancement of soil fertility if the valorization of the side stream of organic matter is balanced with the need of keeping the organic matter in the regenerative systems. Support in policy and investment is vital for the development of infrastructure relating to collection, processing and logistics for the side-stream valorization, in order to insure that circular bio economy policies are coherent with food security, rural livelihoods and ecosystem health.

### 5.3. Socioeconomic, Ethical and Regulatory Dimensions

The growth of alternative proteins is driven by accelerating market growth rates, significant venture capital and corporate investment, and favorable policy discourse, positioning these products as solutions to climate, health, and food security problems [3]. Economic assessments indicate that alternative proteins can create new value chains and generate high-skilled jobs in biotechnology and food processing []. They may also mitigate systemic risks associated with zoonotic diseases and climate-related disruptions in cattle systems [3]. The distribution of benefits and burden, both across the supply chain (including the impacts on farmers, rural communities and laborers in traditional meat industries), raises important questions about justice and transition.

Meat analogs, cultured meat and other alternatives often are promoted for their ability to reduce animal suffering and environmental damage [5]. However, concerns persist about ultra-processing, corporate consolidation, ownership of data and the cultural impacts on traditional eating practices. Regulatory frameworks for the innovative proteins and technologies (e.g., cultured meat, precision fermentation, insect-based foods) typically follow the same standards for novel foods, food safety, and labeling and thus require thorough risk assessments, transparency and clear information on nutritional and compositional data [6]. Discussions on labeling—particularly the use of terms such as “burger,” “sausage,” or “milk” for plant-based products—highlight conflicts between consumer information needs, fair market competition, and the protection of existing industries [6]. These debates underscore the need for evidence-based coherent standards that promote innovation while ensuring consumer confidence.

**Figure 5:**
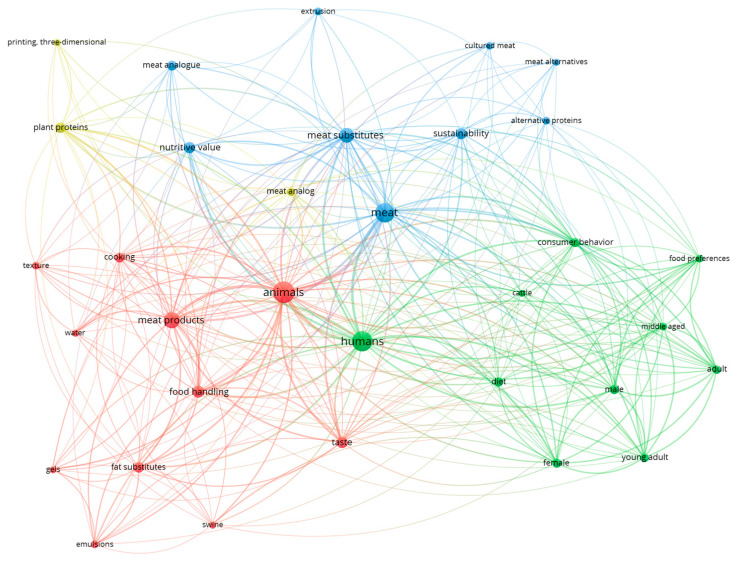
A keyword co-occurrence network, which is generated from the literature published over the past 10 years on challenges to the meat analog industry (derived using the keyword challenges to meat analog industry in PubMed in the literature published in the last 10 years). The colors represent distinct thematic clusters identified in the keyword co-occurrence network, with each cluster grouping related research topics within the field of alternative proteins.

Alternative proteins face interconnected technological, economic, and societal challenges that need to be overcome to make the transition from niche to mainstream protein sources. There is a need for serious attention to critical scale-up impediments and the need for multi-criteria optimization and prioritized research trajectories and frameworks. Moreover, addressing these challenges is highly important, as the increasing number of studies published in recent years, as illustrated in Figure 5, reflects the expanding research landscape in alternative proteins.

## 6. Challenges, Knowledge Gaps, and Future Directions

### 6.1. Technological and Scale-Up Challenges

Economically viable scale-up is a commonly referenced challenge for alternative proteins, impacting plant-based, microbial, insect, and cultured systems uniformly [8]. Numerous promising technologies, such as shear-cell structuring, high-moisture extru-sion variations, and precision fermentation, have been established at laboratory and pilot scales; nevertheless, their transition to industrial scale is hindered by capital expenditures, process intricacy, and ingredient inconsistency. In this review, it is highlighted that alternative proteins usually require extraction, separation, or fermentation stages that increase energy use and costs compared to direct animal products, so it is of great importance to techno-economically optimize these processes. Connecting laboratory, pilot, and industrial scales for structured meat analogs poses unique engineering challenges such as non-linear variation in residence time, heat transfer and shear field effects on texture and product uniformity with increase in equipment size [188]. The redesign of food grade bioreactors, developing serum free cost-effective media, and simplifying downstream purification are highlighted as critical to competitive pricing in the case of fermentation-based and cultured meat [189]. Sector-wide assessments underscore additional supply chain challenges, resulting in limited local fractionation capacity and irregular access to high-quality plant protein isolations, with potential impacts on robustness and reproducibility at scale.

### 6.2. Multi-Criteria Optimization of Next-Generation Products

Recent studies highlight that alternative proteins must be optimized according to multiple criteria and at the same time, i.e., based on sensory quality, nutrition, environmental impact, safety and affordability, rather than focusing on single criteria, e.g., protein content or carbon footprint, only [190]. Many current products still have problems replicating the organoleptic experience of meat, especially in terms of texture and flavor, whilst also meeting expectations for clean labels, low sodium, and better fatty acid profiles. The integration of life cycle thinking into product design can reveal trade-offs; for instance, highly processed ingredients may enhance texture while increasing energy demand and cost.

AI, big data, and omics are increasingly suggested as enablers of such multi-objective optimization. Recent studies on alternative protein development have highlighted the use of AI for ingredient discovery, protein functionality prediction, strain design for precision fermentation, and process-scale-up modeling. In parallel, omics-driven approaches and artificial intelligence (AI)-enabled bioinformatics are being leveraged to predict protein structure, digestibility and allergenicity, which can be used to guide the approaches and engineering for selection of protein sources for both functionality and safety. Combining these tools with detailed process and sensory data sets is considered the path to accelerated and data-driven optimization of formulations and structuring technologies.

### 6.3. Research Priorities and Research Roadmap

Several recent road-mapping exercises/reviews have articulated priority research questions for alternative proteins/meat analogs. Technical roadmaps suggest which bottlenecks to identify, for example, green and high efficiency extraction of low-denatured plant proteins, construction of meat-like multi-dimensional structures beyond the extrusion process, development of stable myogenic cell lines and serum-free media for cultivated meat, and affordable production of growth factors and scaffolds. Reviews of meat analog technologies also call for a better understanding of structure–function relationships in complex protein blends, improved modeling of thermomechanical structuring, and improved integration of fats, fibers, and flavors to more closely approximate whole-muscle meat quality.

**Figure 6:**
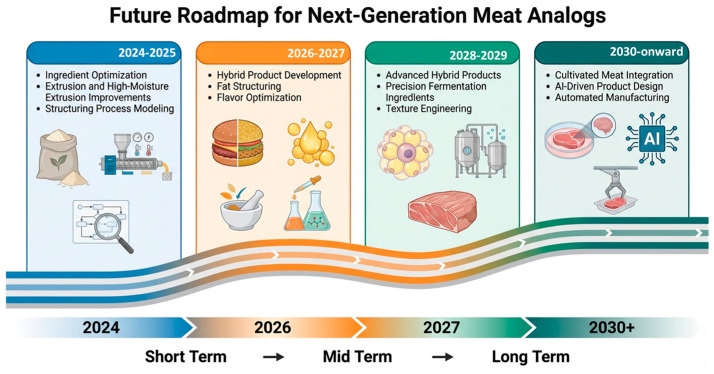
Roadmap for next-generation meat analogs.

At the system level, the importance of policy and economics reports focus on the need for coordination of R&D, infrastructure investments, and enabling regulation to scale production and lower costs, while ensuring fair competition and inclusion of farmers. Priority research directions are scaling-up sources of protein ready for adaptation to local agro-ecologies, designing clean-label binders and gelling systems, creating color and flavor solutions mimicking meat with lesser processing intensity, and producing robust comparative data on the nutritive, health and environmental impacts for the purposes of developing guidelines and standards. Prospects for incorporating different protein categories—plant, microbial, insect and cultivated—into hybrid products and diversified supply chains are generally considered as an important avenue for resilience, so that the complementary strengths of each technology are combined in next-generation meat analogs and other formats. Some main research priorities and development pathways for next-generation meat analogs are summarized in the roadmap presented in Figure 6.

## 7. Conclusions

Alternative proteins and advanced processing technologies are redefining modern food systems by offering viable pathways toward sustainable, resource-efficient, and nutritionally adequate protein production. This review demonstrates that plant, microbial, insect, and cultivated protein sources each provide distinct techno-functional, nutritional, and sustainability advantages; however, their practical application in meat analog systems remains dependent on overcoming key challenges related to texture development, flavor optimization, scalability, and cost.

A critical evaluation of the literature indicates that no single protein source or processing technology is universally optimal. Plant proteins currently dominate due to their availability and established functionality, whereas microbial and cultivated proteins present promising future opportunities despite limitations in cost, regulatory approval, and industrial scalability. Similarly, structuring technologies such as high-moisture extrusion, shear-cell processing, and emerging non-thermal approaches offer different trade-offs in terms of product quality, energy requirements, and feasibility. Therefore, hybrid strategies that combine multiple protein sources and complementary processing techniques represent the most practical approach for achieving desirable sensory, nutritional, and economic outcomes.

Importantly, beyond their structural and nutritional roles, alternative proteins also act as intrinsic sources of antioxidant compounds, including bioactive peptides, polyphenols, carotenoids, and vitamins. These components contribute to oxidative stability through radical scavenging, metal chelation, and synergistic interactions within complex food matrices. The relationship between protein structure, processing conditions, and antioxidant functionality is particularly significant as thermal and mechanical treatments can both enhance and degrade antioxidant activity. Consequently, the integration of antioxidant functionality into ingredient selection, processing design, and product formulation is essential for improving shelf life, color stability, flavor retention, and overall product quality in meat analogs. This highlights the need for integrated design strategies where antioxidant functionality, processing conditions, and protein selection are optimized simultaneously rather than independently.

Despite significant advancements, several knowledge gaps remain. These include the need for a deeper mechanistic understanding of structure–function relationships in complex protein systems, standardized methods for evaluating oxidative stability and antioxidant performance, and more robust comparative data across different protein sources and processing technologies. In addition, sustainability assessments such as life cycle analysis require harmonization, as outcomes are highly sensitive to system boundaries, processing intensity, and energy inputs.

Future research should focus on multi-criteria optimization approaches that integrate sensory quality, nutritional value, oxidative stability, environmental impact, and economic feasibility. Advances in protein engineering, precision fermentation, and AI-driven formulation design offer promising tools to accelerate innovation. Furthermore, the development of clean-label antioxidant strategies, scalable processing technologies, and region-specific product designs will be essential to enhance consumer acceptance and market adoption.

The transition toward next-generation meat analogs will depend on the effective integration of diverse protein sources, advanced structuring technologies, and antioxidant functionality within a system-level framework. Such an approach will be critical to achieving resilient, sustainable, and consumer-acceptable protein solutions for the future.

## Figures and Tables

**Table 1:** Comparison of major alternative protein sources used in meat analogs.

Protein Source	Key Techno-Functional Properties	Sustainability Advantages	Key Limitations	Sources	References
Plant-based proteins	Gelling, emulsification, water/fat binding, fiber formation under shear	Low GHG emissions, scalable, established supply chains	Beany off-flavors, anti-nutritional factors, allergenicity	Soy, pea, wheat gluten, lentils	[23]
Microbial proteins	Natural fibrous texture, umami contribution, emulsification	Minimal land use, high productivity, side-stream utilization	Regulatory approval, consumer perception	Mycoprotein, yeast SCP, microalgae	[24]
Fermentation-derived proteins	Flavor enhancement, color development, binding	Efficient resource use, targeted functionality	Cost, regulatory complexity	Precision-fermented heme, enzymes	[25]
Insect proteins	Emulsification, protein enrichment	High feed efficiency, waste valorization	Consumer acceptance, allergenicity	Mealworm, cricket, BSF larvae	[26]
Cultivated meat	Authentic flavor, species-specific identity	Reduced land-use potential	High cost, scale-up, regulatory barriers	Muscle and fat cells	[27]
Hybrid meat analogs	Improved texture, flavor, nutrition	Cost reduction, scalability	Formulation complexity	Plant + microbial or cultivated	[28]

**Table 3:** Physicochemical and structural quality attributes of meat analogs.

Quality Attribute	Engineering/Processing Strategies	References
Microstructure & Fiber Alignment	High-moisture extrusion, shear-cell processing, and controlled cooling to promote protein alignment	[86]
Porosity & Fat Distribution	Controlled mixing, extrusion structuring, and stabilization of fat emulsions	[101]
Water Holding Capacity (WHC)	Protein gelation, addition of hydrocolloids and dietary fibers to improve moisture retention	[102]
Fat Binding Capacity	Protein–lipid interactions and emulsion stabilization during processing	[103]
Texture Profile (Hardness, Cohesiveness, Chewiness, Springiness)	Adjustment of protein source, extrusion conditions and post-processing (cooling, storage)	[70]
Color Development	Use of natural colorants and controlled Maillard reactions during thermal processing	[104]
Matrix Integrity	Use of binders such as proteins, starches, and fibers to strengthen the protein network	[58]
Gelation & Thermal Stability	Hydrocolloid addition (e.g., methylcellulose, carrageenan) and heat-induced gel formation	[105]

**Table 4:** Nutritional and health considerations of meat analogs in comparison to conventional meat.

Attribute	Meat Analogs	Conventional Meat	Reference
Protein content	High (formulation-dependent)	High	[123]
Fat profile	Higher unsaturated fats	Higher saturated fats	[124]
Dietary fiber	Present	Absent	[125]
Sodium	Often elevated	Moderate	[126]
Micronutrients	Fortified (B12, Fe, Zn)	Naturally present	[119]

**Table 5:** Role of antioxidants in improving oxidative stability, shelf life, and color retention in meat analogs.

Antioxidant Source	Key Bioactive Compounds	Mechanism of Action	Oxidative Stability	Color Retention	Effective Concentration/Dose
Green Tea Extracts	L-theanine, Tannins	Reduces protein deterioration & lipid oxidation	Stable over 28-day storage [136]	Improved emulsion stability & texture	1.0% (GEE optimal)
Olive Mill By-products	Oleacein, Verbascoside, Hydroxytyrosol	Maintains phenolic concentration, inhibits microbial growth	High & stable throughout shelf life [137]	Maintained color during storage	30 g/kg powder
Carrot & Tomato Powder	Polyphenols, Flavonoids	Increases TPC, prevents lipid oxidation during cooking [138]	Increased antioxidant stability	Tomato > carrot (color stability)	0.025–2.5% *w*/*w*
Ginseng Extract	Polyphenols (FRAP, DPPH)	FRAP & DPPH scavenging, controls TBARS & LPO [155]	Reduced TBARS & LPO after 30 days	pH decrease, color parameters affected	1% + (ground beef)
*Epilobium angustifolium* Extract	Gallic acid, Phenolics	Antioxidant & antimicrobial, reduces MDA formation [156]	Moderate (prooxidant at >9 g)	Color effects at different concentrations	1–9 g (optimal ~3–9 g)
*Litsea cubeba* & Cinnamon Essential Oils	Terpenoids, Phenolics	Inhibits *E. coli* & *S. aureus*, reduces TBARS [139]	Significant reduction in TBARS	Improved color & pH balance	Nanoemulsion coating applied
Rosemary, Oregano, Thyme, Clove Oils	Phenolics, Terpenoids, Flavonoids	Free-radical scavenging, prevents oxidation [140]	Extended shelf life, controlled spoilage	Prevents discoloration, maintains L* (Lightness), a* (Redness), b* (Yellowness)	0.025–2.5% *w*/*w*
Liposome-Based GA/Res System	Gallic Acid + Resveratrol	Suppresses LOOH & MDA, reduces B(a)P formation [141]	Effective LOOH & MDA suppression	Delays browning, maintains color	GA/Res-L encapsulated
Adzuki Bean Protein	Polyphenols, Natural colorants	Radical scavenging, intrinsic stability [147]	Stable for 1 year at 25 °C	Natural red hue, intrinsic stability	Protein isolate 84.5%
Berry Pomace	Polyphenols, Dietary fiber	Enhanced antioxidant capacity & color stability [148]	Enhanced shelf stability	Natural colorants, enhanced appeal	Direct addition or extract
Seaweed (40% addition)	Polyphenols, Polysaccharides	Radical scavenging & water management [157]	Significant oxidative damage reduction	Restricted water migration, color preserved	40% seaweed addition optimal
Glucose Oxidase + Tamarind Gum	Enzymatic + Polymer network	Cross-linking + steric stabilization [142]	Excellent protein & lipid protection	Minimal color variation, texture maintained	GO + TG combination

**Table 6:** Environmental performance indicators.

Product Type	GHG Emissions	Land Use	Water Use	Relative Impact vs. Beef	References
Plant-based meat	Lower	Lower	Lower	Substantially lower	[5,182]
Fermentation-derived protein	↓ 60–70%	Minimal	Lower	Substantially lower	[6,183]
Cultivated meat	Variable	Potentially higher than other meat analogs but lower than conventional beef	Variable	Energy-dependent	[8]
Conventional beef	High	Very high	High	Reference	[184]

↓ reduced or decreased

## Data Availability

No new data were created or analyzed in this study. Data sharing is not applicable to this article.
